# Chiral Antioxidant-based Gold Nanoclusters Reprogram DNA Epigenetic Patterns

**DOI:** 10.1038/srep33436

**Published:** 2016-09-16

**Authors:** Yue Ma, Hualin Fu, Chunlei Zhang, Shangli Cheng, Jie Gao, Zhen Wang, Weilin Jin, João Conde, Daxiang Cui

**Affiliations:** 1 Institute of Nano Biomedicine and Engineering, Shanghai Engineering Center for Intelligent Diagnosis and Treatment Instrument, Department of Instrument Science and Engineering, Key Lab. for Thin Film and Microfabrication Technology of Ministry of Education, School of Electronic Information and Electronic Engineering, Shanghai Jiao Tong University, Shanghai 200240, China; 2 National Center for Translational Medicine, Shanghai Jiao Tong University, 800 Dongchuan Road, Shanghai 200240, PR China; 3 School of Life Sciences and Biotechnology, Shanghai Jiao Tong University, Shanghai 200240, China; 4 Department of Experimental Surgery, Tangdu Hospital, Fourth Military Medical University, Xi’an 710038, China; 5 Massachusetts Institute of Technology, Institute for Medical Engineering and Science, Harvard-MIT Division for Health Sciences and Technology, Cambridge, Massachusetts, USA; 6 School of Engineering and Materials Science, Queen Mary University of London, London, UK

## Abstract

Epigenetic modifications sit ‘on top of’ the genome and influence DNA transcription, which can force a significant impact on cellular behavior and phenotype and, consequently human development and disease. Conventional methods for evaluating epigenetic modifications have inherent limitations and, hence, new methods based on nanoscale devices are needed. Here, we found that antioxidant (glutathione) chiral gold nanoclusters induce a decrease of 5-hydroxymethylcytosine (5hmC), which is an important epigenetic marker that associates with gene transcription regulation. This epigenetic change was triggered partially through ROS activation and oxidation generated by the treatment with glutathione chiral gold nanoclusters, which may inhibit the activity of TET proteins catalyzing the conversion of 5-methylcytosine (5mC) to 5hmC. In addition, these chiral gold nanoclusters can downregulate TET1 and TET2 mRNA expression. Alteration of TET-5hmC signaling will then affect several downstream targets and be involved in many aspects of cell behavior. We demonstrate for the first time that antioxidant-based chiral gold nanomaterials have a direct effect on epigenetic process of TET-5hmC pathways and reveal critical DNA demethylation patterns.

Gold nanoparticles have been extensively exploited for potential biomedical applications due to their extraordinary optical and electronic properties, presenting several shapes such as nanorods, nanoshells, nanospheres and nanocages^1^,^2^. Composed of a few to a hundred atoms, the size of gold nanoclusters (AuNCs) is comparable to the *de Brogliewavelength* at the *Fermi* level, resulting in molecule-like properties including discrete electronic states and size-dependent fluorescence. High stability, biological compatibility, diverse sizes and easy surface functionalization have made AuNCs ideal platform for biomedical applications, such as cancer diagnostics, therapy and bioimaging^3^,^4^,^5^,^6^,^7^,^8^,^9^,^10^,^11^,^12^.

Although the cytotoxicity of nanoparticles have been extensively assessed^13^,^14^,^15^, the underlying mechanism of toxicity induced by nanoparticles remains to be fully elucidated. It has been reported that the toxicity of gold nanoparticles is dependent on the chemical composition of the surface ligands^16^ and size of the nanoparticles^13^. However, the knowledge of how cells interact with this class of ultra-small nanoparticles remains underexplored, especially the effect of AuNCs on epigenetics regulation.

Epigenetics refers to the changes of gene expression caused by external or environmental factors other than changes in the DNA sequence, some of which are heritable^17^,^18^,^19^. DNA methylation is one of the main epigenetic modifications. Perturbed DNA methylation patterns are associated with many human diseases such as cancer^20^, neurological disorders^21^ etc. The enzymes that catalyze DNA methylation such as DNMTs have been well studied. The ten-eleven translocation (TET) family of 5-mC hydroxylases includes TET1, TET2 and TET3. The TET proteins have been shown to function in transcriptional activation and repression (TET1), tumor suppression (TET2), and DNA methylation reprogramming processes (TET3). The discovery of TET proteins has shed light on the DNA demethylation mechanism, which is an exciting advance in the epigenetic field. TET proteins are capable of catalyzing the oxidation of 5-methylcytosine (5mC) into 5-hydroxymethylcytosine (5hmC) in mammalian DNA^22^,^23^. 5hmC is an important marker in epigenetics, which is mainly involved in up-regulation of gene expression associated with cell differentiation, neuronal development and aging^21^.

Among the thiolate protected Au NCs, chiral L-glutathione (i.e. L-GSH; N-γ-glutamyl-cysteinyl-glycine), a naturally occurring tripeptide, which intensively exists in living cells to maintain the reducing state of cellular potential and to protect biological molecules against oxidative damage, was mainly used as the thiolate ligand for the synthesis of AuNCs^24^. It is evident that the ligand shell can tune the (bio)chemical properties and exert a tremendous influence on the applications of thiolate-protected gold nanoclusters. So we choose L-GSH and D-GSH as the capping ligands of Au NCs.

Here we showed for the first time that antioxidant-based chiral AuNCs capped with GSH enantiomers - L-GSH or D-GSH have a strong effect on 5hmC levels and gene expression regulation (specially the L-GSH AuNCs), which emphasizes the important effect on epigenetics by nanomaterials. These antioxidant-based chiral AuNCs can down-regulate the mRNA expression level of TET1 and TET2, and also induce the aggregations of TET proteins through oxidation of their catalytic domains. These alterations can result in the decreasing of the catalytic activities of TET proteins and the subsequent global decline of 5hmC levels, which leads to gene expression changes that are involved in cell adhesion, migration, proliferation, differentiation and cell apoptosis. This demethylation process is necessary for epigenetic reprogramming of genes and is also directly involved in many important disease mechanisms such as tumor progression.

## Characterization of glutathione-based chiral gold nanoclusters

**Figure 1:**
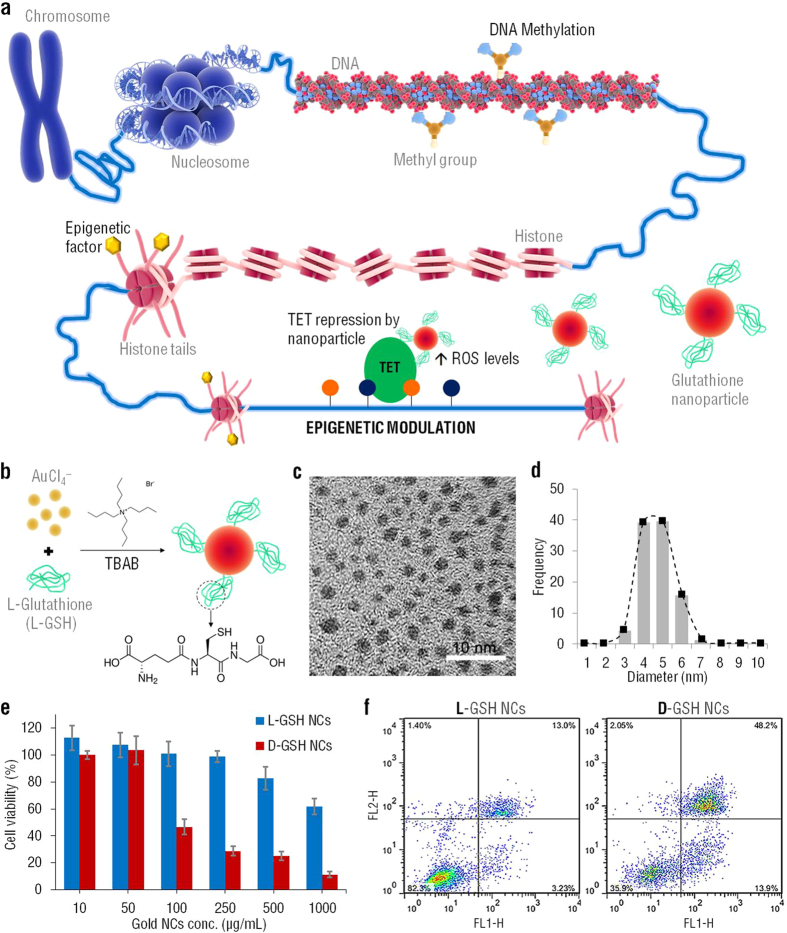
Characterization of L-GSH capped AuNCs and evaluation of its cytotoxicity, when compared with its enantiomer D-GSH capped AuNCs. (**a**) Regulation of epigenetic events associated with DNA demethylation processes, which is the removal of a methyl group, using antioxidant-based chiral AuNCs capped with enantiomers of L-GSH or D-GSH (N-γ-D-glutamyl-D-cysteinyl-glycine). (**b**) Schematic of the synthesis of AuNCs@L-GSH.TBAB: tetrabutylammonium borohydride. (**c**) High-resolution TEM image of AuNCs@L-GSH. Scale bar = 10 nm. (**d**) Size distribution of AuNCs@L-GSH. (**e**) Cell viability (MTT assay) for MGC-803 cells treated with AuNCs@L-GSH and AuNCs@D-GSH, the data were normalized to control group (cells cultured in normal media). (**f**) Flow cytometry analysis of apoptosis/necrosis in MGC-803 cells after exposure to 100 μg/ml of AuNCs@L-GSH and AuNCs@D-GSH for 24 h. FL1-H axis represents cells stained with Annexin-V FITC dye. FL2-H represents cells stained with propidium iodide (PI) dye. All experiments were done in triplicates and errors reported as standard deviation (s.d.).

Antioxidant-based chiral AuNCs capped with enantiomers of L-GSH or D-GSH (N-γ-D-glutamyl-D-cysteinyl-glycine) were used to actively regulate epigenetic events associated with DNA demethylation processes, which is the removal of a methyl group (Fig. 1a). The demethylation process is necessary for epigenetic reprogramming of genes and is also involved in several key disease mechanisms such as cancer progression. Commonly the removal of 5-methylcytosine occurs via the sequential modification of cytosine bases that have been converted by TET enzyme-mediated oxidation. Here we designed smart glutathione-based chiral gold nanoclusters to tackle this mechanism and reprogram key driver genes involved in cell adhesion, migration, proliferation, differentiation and cell apoptosis. The synthesis of AuNCs@L-GSH was performed using TBAB: tetrabutylammonium borohydride (Fig. 1b). High-resolution TEM images (Fig. 1c) and size distribution (Fig. 1d) of AuNCs@L-GSH revealed that these nanoclusters present a well disperse distribution with approximately 4–5 nm in diameter.

Next, the biocompatibility of the glutathione-based chiral gold nanoclusters designed here was evaluated in order to define the best nanoformulation to evaluate and reprogram epigenetics patterns. Cytotoxicity of L- and D-GSH-capped AuNCs was determined using the MTT colorimetric assay (Fig. 1e). AuNCs@L-GSH displayed minimal toxicity at dose up to 250 μg/ml against human gastric cancer cell line (MGC-803), with an IC_50_ (half-maximum inhibitory concentration) of 1068.6 ± 59.2 μg/ml (Fig. 1e). Conversely, AuNCs@D-GSH induced a significant decrease in cell viability with doses up to 1000 μg/ml. The IC_50_ values for the viability of cells treated with AuNCs@D-GSH is 87.3 ± 3.4 μg/ml. Furthermore, the apoptosis mechanisms of MGC-803 cells treated with AuNCs@L-GSH and AuNCs@D-GSH were evaluated and compared using an Annexin V-FITC/Propidium Iodide double staining method. The percentage of cells in early and late apoptotic, as well as necrotic stages in MGC-803 cells exposed to 100 μg/ml of AuNCs@L-GSH and AuNCs@D-GSH for 24 h was quantified by flow cytometry (Fig. 1f). The majority of cells undergo apoptosis (approximately 14% for early apoptosis, 48% for late apoptosis) rather than necrosis when exposed to AuNCs@D-GSH, while only around 13% and 3% of cell death was due to early and late apoptosis when exposed to AuNCs@L-GSH (Fig. 1f). These results are consistent with the decrease in cell viability of MGC-803 cells treated with AuNCs@D-GSH, when compared with AuNCs@L-GSH.

**Figure 2:**
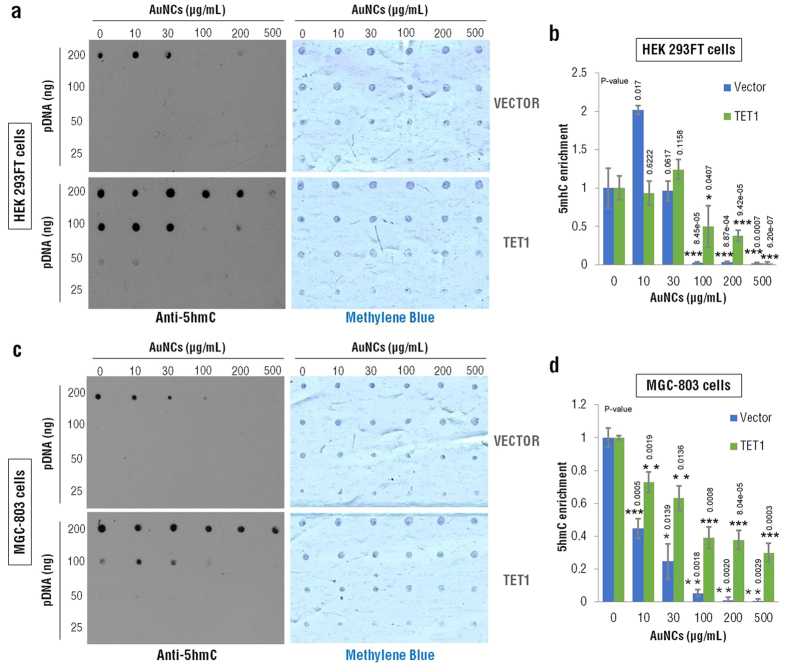
Dot blot for 5hmC of HEK 293FT cells and MGC-803 cells treated with indicated concentration of AuNCs@L-GSH. HEK 293FT cells and MGC-803 cells were transfected with a control vector and with TET1 expression plasmids and then treated with increasing concentrations of AuNCs@L-GSH for 48 h. Total DNA was harvested and Dot blot was conducted for 5hmC in HEK 293FT cells (**a**) and MGC-803 cells (**c**). The nylon membranes were stained with methylene blue for DNA as loading control. The densitometry quantification was analyzed as in (**b**,**d**) for HEK 293FT cells and MGC-803 cells, respectively. Data are expressed as mean ± SEM. Statistical comparisons between groups were conducted by unpaired Student’s t-test. P-values were shown in the figure. *indicates p < 0.05; **indicates p < 0.01; ***indicates p < 0.001. The value of p < 0.05 was considered to be significant.

It is expected that the toxicity of nanoparticles has an intimate relationship to their uptake and intracellular distribution. The nanoparticle size, surface area, and surface functionalization are major factors that influence toxicity^25^,^26^. Although the results in Fig. 1e show that both L-GSH capped AuNCs and D-GSH capped AuNCs cause cytotoxicity in MGC803 cells at higher concentrations, there was little cytotoxicity at a low concentration for AuNCs@L-GSH. Therefore, since the AuNCs@L-GSH used in this study showed less cytotoxic than the AuNCs@D-GSH, the enantiomer L-GSH was the one used in the following epigenetic studies. The AuNCs@L-GSH were synthesized using tetrabutylammonium borohydride(TBAB)^27^ (Fig. 2c) and exhibit a spherical shape (Fig. 2d) and an outstanding size uniformity (Fig. 2e). The AuNCs@L-GSH displayed good bio-compatibility and were proved to be stable in PBS and DMEM at 37 °C for 24 h. Microscopic observations of monolayer cultured MGC-803cells and HEK 293FT cells did not show obvious morphological changes comparing to the controls until the final concentration up to 1,000 μg/mL. No toxicity was observed when the MGC-803 cells and HEK 293FT cells were treated with a final concentration up to 500 μg/mL of AuNCs@L-GSH.

## Glutathione-based nanoclusters reduce 5-Hydroxymethylcytosine levels

In order to evaluate if glutathione-based chiral gold nanoclusters can alter 5-Hydroxymethylcytosine (5hmC) levels, HEK293FT cells (Fig. 2a) and MGC-803 cells (Fig. 2c) were transfected with a control or a TET1 expression plasmid respectively. Then the cells were treated by adding increasing doses of AuNCs@L-GSH during 48 h. Total DNA was extracted and a dot blot assay was performed to analyze possible changes in 5hmC levels. The nylon membranes were stained with methylene blue for total DNA as a loading control (Fig. 2a,c). We found that, using densitometry quantification (Fig. 2b,d), the levels of 5hmC were significantly down-regulated (p < 0.01) by exposure to 100 μg/mL of AuNCs@L-GSH in both cell lines, when comparing cells transfected with a control or a TET1 expression plasmid. In addition, the 5hmC levels decreased gradually with increasing AuNCs@L-GSH concentrations (p < 0.01). These results indicate that chiral gold nanoclusters may be related with a reduce 5-Hydroxymethylcytosine (5hmC) levels, which is known as the “Sixth base” of DNA that infiltrates into the major groove of DNA and regulate transcription.

## TET proteins catalyze conversion of 5mC to 5hmC

**Figure 3:**
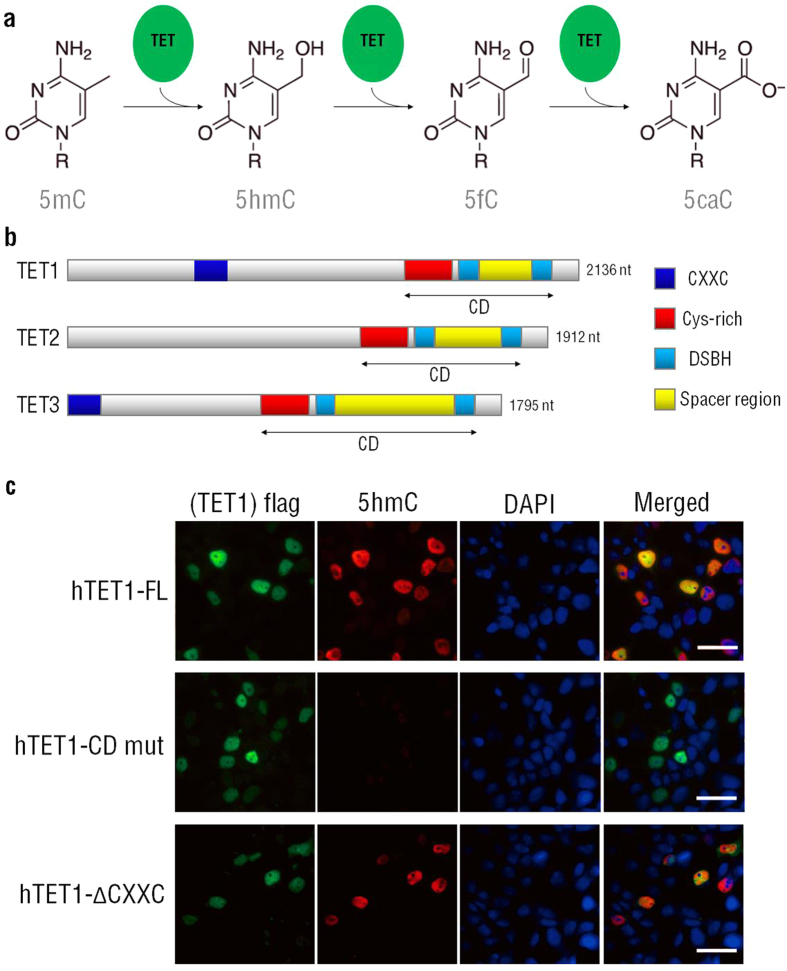
TET proteins catalyze demethylation of 5mC. (**a**) Illustration of TET protein catalyzing 5mC, 5hmC, 5fC and 5caC conversion. (**b**) Schematic of TET proteins structure. (**c**) Immunostaining of TET1 and 5hmC in HEK 293FT ectopic expressing Flag tagged TET1-FL (TET1 full-length), TET1-ΔCXXC (TET1 mutation with CXXC domain deletion) and TET1-CD mut (TET1 with CD domain mutation). DAPI stains the nucleus. Scale bar = 100 μm.

TET1, TET2 and TET3 are a family of Fe^2+^ and α-ketoglutarate (α-KG) dependent dioxygenases, which can utilize molecular oxygen to transfer a hydroxyl group to 5mC to generate 5hmC^22^,^23^,^28^,^29^. 5hmC is the first oxidative product in the multistep-reaction of 5mC demethylation catalyzed by TET proteins. Studies have shown that TET proteins can further catalyze the sequential conversion of 5hmC to 5-formylcytosine (5fC) and 5-carboxylcytosine (5caC), and these 5mC derivatives can be further modified by thymine-DNA glycosylase (TDG) and excised by base excision repair or by replication-dependent dilution, resulting in DNA demethylation^30^,^31^ (Fig. 3a).

Human TET1 and TET3 proteins contain an amino-terminal CXXC domain and a carboxy-terminal catalytic domain (CD domain), which consist of a Cys-rich domain and a double-stranded β-helix (DSBH) domain. It is worth noting that CXXC domain of TET2 is lost during evolution^32^,^33^ (Fig. 3b).

We next validated that CD domain of TET1 can catalyze the conversion of 5mC to 5hmC (Fig. 3c). Ectopic expression of TET1 full-length protein in HEK 293FT cells causes an increase in 5hmC levels, which is abrogated by mutation of CD domain (Fig. 3c), whereas the ΔCXXC mutant still possesses the dioxygenase activity.

## Glutathione-based nanoclusters regulate gene expression associated with 5hmC and TET

**Figure 4:**
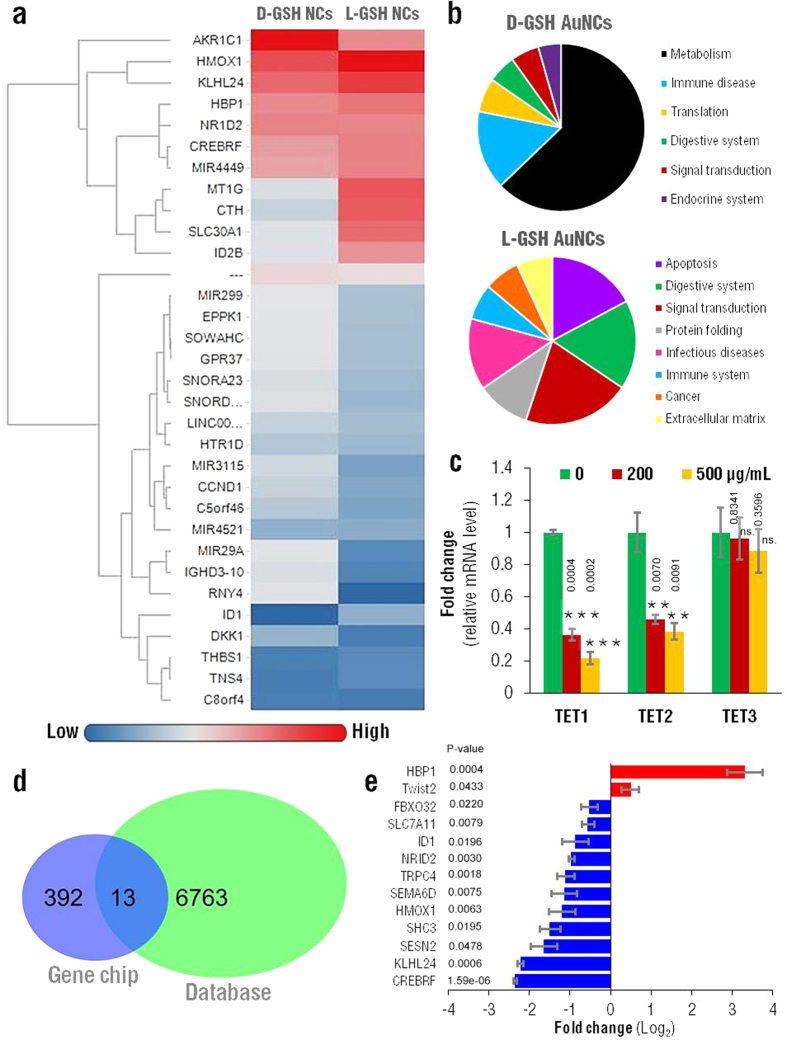
Glutathione-based chiral gold nanoclusters regulate gene expression associated with 5hmC and TET. MGC-803 cells were treated with indicated concentrations of AuNCs@L-GSH and AuNCs@D-GSH for 12 h, the cells cultured in normal media were denoted as control group. (**a**) Heat-map for Gene Expression Array of cells treated with 100 μg/mL AuNCs@L-GSH and AuNCs@D-GSH. (**b**) Pathway analysis based on KEGG of significantly altered genes after treatment with AuNCs@L-GSH and AuNCs@D-GSH. (**c**) qRT-PCR of relative *TET* mRNA levels for AuNCs@L-GSH treated samples comparing to control. *ACTB* (beta-actin) was served as a reference gene. Data are expressed as mean ± SEM. Statistical comparisons between groups were conducted by one-way ANOVA followed by a Newman-Keuls comparison test. P-values were shown in the figure. *indicates *p* < 0.05; **indicates *p* < 0.01; ***indicates *p* < 0.001. The value of *p* < 0.05 was considered to be significant. (**d**) Venn diagram shows the intersectional genes associated with 5hmC, *TET1* and *TET2* from relevant database and gene chips for AuNCs@L-GSH treated samples. Databases refer to the gene chip data from references containing 5hmC related differentially expressed genes^35^ (NCBI GEO accessions: GSE38118) combined with TET related genes^36^ (NCBI GEO accessions: GSE50198 and GSE50200). (**e**) qRT-PCR to validate the gene expression changes in (**a**).

The previous experiments showed that AuNCs@L-GSH treatment reduces global 5hmC in MGC-803 and HEK 293T cells. And we validated the conversion of 5mC to 5hmC is catalyzed by TET protein, which is consistent with the previously reported^22^. Since the association of AuNCs@L-GSH in *TET* mRNA expression and associated genes is not clear, these cells were treated with 0, 200 and 500 μg/mL AuNCs@L-GSH for 24 h and the genetic expression profile of treated cells was performed. As the gene expression profile of a cell determines its phenotype and its response to various factors and therapies, a genome wide expression array (human transcriptome array Affymetrix Human HTA2.0) was performed in cells treated with AuNCs@D-GSH and AuNCs@L-GSH and in cells with no treatment (control). Differentially expressed genes were screened out through fold change. The threshold set for up- and down-regulated genes was a fold change greater than or equal to 1.5. There were 392 differentially expressed genes in total in combination of AuNCs@L-GSH vs. control and AuNCs@D-GSH vs. control (Fig. 4d).

For gene expression heat-maps (Fig. 4a) the average expression across three independent replicates in each condition basing on the probes with the maximum signal intensity per gene was calculated. Heat maps were created using Genesis 1.7.639 based on Pearson correlation coefficients of each replicate per condition. Pathway analyses were performed using Gene Set Enrichment Analysis tool v2.0.13 (GSEA2-2.0.13). The analyses were based on differentially expressed genes in each group. Differential expression was defined as multiple testing adjusted p values smaller than or equal to 0.05 and fold change greater than or equal to 1.5-fold by probes with the maximum intensity in each gene. A clustergram of genes that are differentially regulated by application of AuNCs@D-GSH vs. control and for AuNCs@L-GSH vs. control was built (Fig. 4a) as previously described^34^. The data were analyzed by unsupervised hierarchical clustering, which revealed that the two treatment groups had distinct gene expression profiles. Using a threefold change relative to the control group (no treatment) as a criterion for differential expression, numerous genes were extracted from the administration of the two different nanoclusters (AuNCs@D-GSH and AuNCs@L-GSH).

Detailed Kyoto Encyclopedia of Genes and Genomes (KEGG) pathway analysis was used to identify the molecular pathways and describe the biological processes of the transcript profiling data. Based on the Gene Ontology (GO) analysis, the GO terms of “biological process” that were significantly over-represented in each cluster of the heat maps are shown in Fig. 4b. The significant GO terms of the altered genes for cells treated with AuNCs@D-GSH belong to multiple pathways mainly related with metabolism, immune disease and translation processes. However, for cells treated with AuNCs@L-GSH molecular pathways were related mainly with apoptosis, signal transduction and protein folding. These observations indicate that the characteristics of the upregulated genes and downregulated genes resulting from the different treatments were completely different.

In order to evaluate the effect of AuNCs@L-GSH, cells were then harvested and total mRNA was extracted, followed by RT-qPCR to test *TET* mRNA levels. The results show dramatic decline in *TET1 (p* < 0.001) and *TET2 (p* < 0.01) mRNA levels, whereas *TET3* mRNA presents little changes, suggesting that AuNCs@L-GSH mainly affect the mRNA of *TET1* and *TET2* genes (Fig. 4c).

Previous studies prove that TET proteins play a key role in regulating gene transcription, embryonic development and tumorigenesis. The molecular events involved in changing TET activity may be associated with tumorigenesis by regulating gene expression. Our results showed that AuNCs@L-GSH mainly affected *TET1* and *TET2* mRNA, causing a dramatic decline in global 5hmC levels. 5hmC is enriched at both the start sites of actively transcribed genes and extended promoter regions of *Polycomb*-repressed genes. We then screen and cross-referenced several genes associated with 5hmC, and TET1 or TET2 from databases and the gene chips (from Fig. 4) or previous published papers^27^ to validate their expression changes (Fig. 4d). The databases contain the gene chip data from references containing 5hmC related differentially expressed genes^35^ (NCBI GEO accessions: GSE38118) and TET related genes^36^ (NCBI GEO accessions: GSE50198 and GSE50200).

The qRT-PCR results show that *TWIST2* and *HBP1* were up-regulated, whereas *TRPC4*, SH3, *CREBRF, FBXO32, HMOX1, KLHL24, NRID2, SESN2, SEMA6D*, SCLC7A11 and *ID1*were down-regulated in AuNCs@L-GSH treated samples (Fig. 4e).

*HBP1* was up-regulated through AuNCs@L-GSH treatment, which encodes a transcriptional repressor with a role in cell cycle arrest^37^, suggesting that AuNCs@L-GSH may regulate cell cycle. CREBRF (CREB3 regulatory factor) has been reported to negatively regulate the endoplasmic reticulum (ER) stress response (or unfolded protein response)^38^. In the lumen of ER, unfolded proteins may cause stress response of the ER which is initially for damage repair but can eventually trigger cell death if ER dysfunction is severe^39^. Actually, in nanogold particles treated human chronic myelogenous leukemia cells, ER stress responses was elicited through biology analysis of the proteomic data^40^. *ID1* is a member of *ID* genes, which encode transcription factors that play important roles in regulation of cell adhesion, migration, growth, proliferation, differentiation and tumorigenesis^41^,^42^,^43^,^44^. It has been reported that *ID* genes expression were down-regulated both in cell lines and in the liver tissues of mice treated with iron nanoparticles^45^.

## Glutathione-based nanoclusters cause TET proteins aggregation

**Figure 5:**
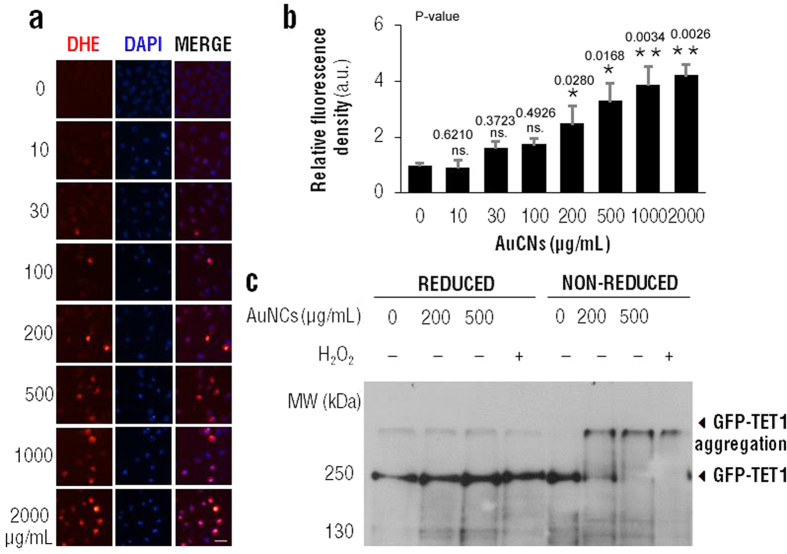
AuNCs@L-GSH cause TET1 protein aggregation. (**a**)MGC-803 cells were treated with indicated concentrations of AuNCs@L-GSH for 24 h. DHE staining was performed to label the ROS levels of MGC-803 cells. (**b**) Densitometry quantification of cell fluorescence stained by DHE in (**a**). Data are expressed as mean ± SEM. Statistical comparisons between groups were conducted by unpaired Student’s *t*-test. P-values were shown in the figure. *indicates *p* < 0.05; **indicates *p* < 0.01. The value of *p* < 0.05 was considered to be significant. (**c**) HEK 293FT cells were transfected with GFP tagged TET1 expression plasmid and then treated with 0, 200 and 500 μg/mL AuNCs@L-GSH respectively for 12 h. Then redox-western blot was performed to detect the GFP-TET1 in reduced and non-reduced condition. Treatment with 1 mM H_2_O_2_ for 5 min was chosen as a positive control.

As we have discussed above, TET proteins belong to a class of α-KGand Fe^2+^-dependent dioxygenases which catalyze an oxidative process. There is a long-standing view that nanoparticle triggered ROS production is one of the principal mechanisms of cytotoxicity. The intracellular ROS levels may also affect TETs activity through oxidation effects. In order to evaluate if glutathione-based chiral gold nanoclusters could generate ROS, Dihydroethidium (DHE) dye staining and cellular uptake was used (Fig. 5a). MGC-803 cells were treated with increasing concentrations of AuNCs@L-GSH for 12 h, and then DHE staining was performed to detect ROS levels. We found that, using fluorescent densities quantification, the ROS levels gradually increase as the concentration of AuNCs@L-GSH rises up, exhibiting significant differences (*p* < 0.05) when the concentration reached 200 μg/mL (Fig. 5b). Next, to validate if AuNCs@L-GSH could also affect TET1 proteins through generating ROS a redox-western blot assay was performed. HEK 293FT cells were transfected with GFP-TET1 expression plasmid^46^,^47^ and treated with 0, 200 and 500 μg/mL AuNCs@L-GSH for 12 h respectively, and 1 mM H_2_O_2_ treatment as positive control. We found that the TET1 protein complex formation occurs under non-reducing conditions as well as with H_2_O_2_ treatment (Fig. 5c), suggesting that AuNCs@L-GSH could induce TET protein complex formation in non-reducing conditions, and then affect their enzymatic activities through ROS generation.

## Glutathione-based chiral gold nanoclusters trigger intracellular ROS generation

**Figure 6:**
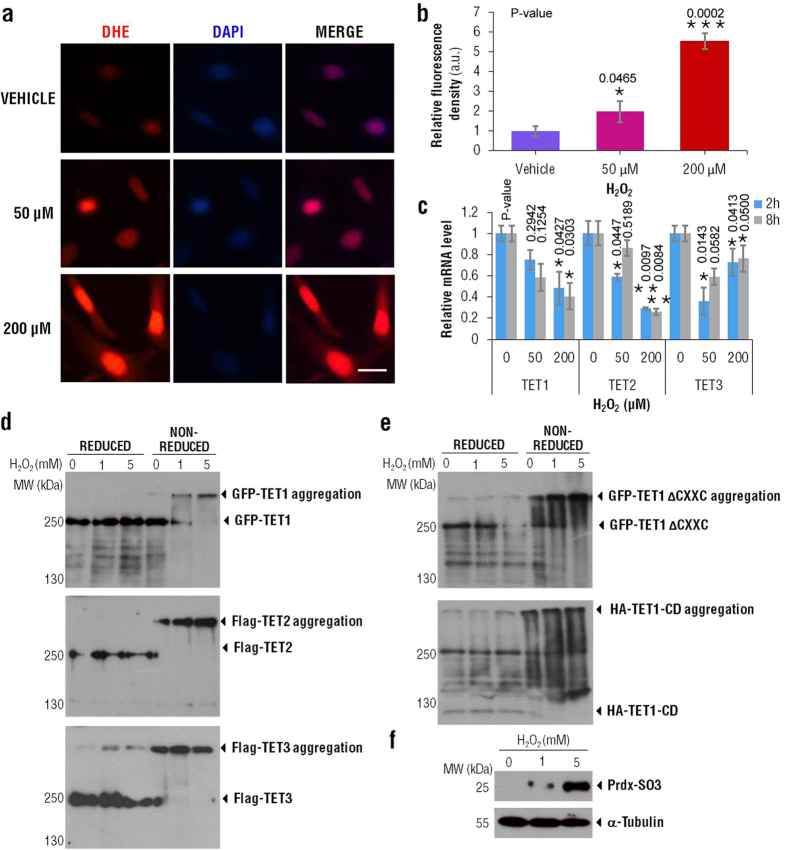
ROS regulates *TET* mRNA expression and TET protein activities. (**a**) DHE staining to evaluate ROS levels in MGC803 cells treated with indicated concentrations of H_2_O_2_. Scale bar = 100 μm.(**b**) Relative fluorescent density of cells stained by DHE in (**a**). Statistical comparisons between groups were conducted by unpaired Student’s t-test. P-values were shown in the figure. *indicates *p* < 0.05; ***indicates *p* < 0.01. (**c**) qRT-PCR was performed to test relative *TET* mRNA expression of MGC-803 cells treated with indicated concentration of H_2_O_2_. *ACTB* was chosen as a loading control. Data are expressed as mean ± SEM. Statistical comparisons between groups were conducted by one-way ANOVA followed by a Newman-Keuls comparison test. P-values were shown in the figure. *indicates *p* < 0.05; **indicates *p* < 0.01. The value of *p* < 0.05 was considered to be significant.(**d**) HEK 293FT cells were transfected with GFP-tagged TET1, Flag-tagged TET2 and Flag-tagged TET3 expression plasmid respectively and cultured for 24 h, then the cells were treated with indicated final concentrations of H_2_O_2_ for 5 min. Cells were lysed in reduced and non-reduced conditions followed by western blot analysis. Molecular weights were determined by a simultaneously run protein ladder. (**e**) HEK 293FT cells ectopic expressing GFP-tagged TET1-ΔCXXC (TET1 mutation with CXXC domain deletion) and HA-tagged TET1-CD (TET1 mutation with only CD domain) were treated with H_2_O_2_ at indicated concentrations for 10 min respectively. Lysates were analyzed by western blot with GFP and HA antibody under reducing or non-reducing conditions. (**f**) The oxidative stress induced by H_2_O_2_ was indicated by Prdx-SO_3_ level as a positive control.

Reactive oxygen species refer to a group of chemically reactive molecules such as superoxide anion (O_2_•−), hydroxyl radicals (•OH) and hydrogen peroxide (H_2_O_2_), that is continually generated through aerobic cellular metabolism and may function as signaling molecules, regulating cell vitality, proliferation, migration and differentiation^48^. However, excess ROS may impair cellular functions. We have previously proved that GSH capped AuNCs can induce intracellular ROS generation previously^49^. In the present study, we used H_2_O_2_ treatment as a positive control to study the ROS regulation on TET proteins and mRNAs. Cellular ROS levels were detected with DHE. Cultured MGC-803 cells were treated with 50 μM and 200 μM H_2_O_2_ for 0.5 h, and then were probed with DHE to visualize ROS levels. Epi-fluorescence images displayed that cellular fluorescent density is positively correlated with the amount of H_2_O_2_ added (Fig. 6a,b). Next, we examined *TET* mRNA expressions and found that the levels of *TET* mRNA were significantly decreased (*p* < 0.05) with increasing H_2_O_2_ concentrations (Fig. 6c), suggesting that TET protein have a decreased activity under oxidative stress, probably via inhibition of demethylase activity.

Redox western blots were then performed to examine the forms of TET proteins with a fixed concentration of H_2_O_2_ treatment under reduced and non-reduced conditions, respectively. We found that TET1,TET2 and TET3 proteins show electrophoretic mobility shift in non-reduced conditions, suggesting that the TET proteins can form large aggregations (termed TET1 complex, TET2 complex and TET3 complex) mediated by oxidation processes (Fig. 6d).

Considering that the TET proteins own a Cysteine-rich domain in their C-terminal, which play essential roles in their catalytic activities, we speculate that oxidative aggregation may result from oxidation in cysteine thiol groups (R-SH), and further formation of inter- or intra-molecular disulfides between other thiol groups. To test our hypothesis, HEK 293FT cells were transfected with two kinds of TET1 truncation, TET1-ΔCXXC (CXXC domain deletion) and TET1-CD (only CD domain), and treated with a fixed concentration of H_2_O_2_. Both TET1-ΔCXXC and TET1-CD truncations can form aggregations under non-reducing conditions, indicating that the CD domain of TET1 was involved in the formation of larger TET1 protein complex (Fig. 6e). The intracellular ROS levels were indicated by the positive control Prdx-SO_3_^50^ (Fig. 6f). Since CD domains of TET proteins are essential for the enzymatic activities, large TET protein complex caused by ROS-induced excessive oxidation in CD domain may impair their function, which decreases the global level of 5hmC in the cells.

Although some potential functions of DNA methylation have been demonstrated already in previous studies, numerous questions remain in terms of unveiling the role of 5hmC. Here we prove that 5hmC serves both as an intermediate of DNA demethylation as well as a stable epigenetic marker.

## Conclusions

In the present study, we first demonstrated that AuNCs@L-GSH not only cause cytotoxicity at high doses, but also change the 5hmC levels at non-cytotoxic doses. As corroborated by our data, 5hmC can influence both long and short-term regulation of gene expression, which will likely have biological significance *in vivo*. We then found that the non-cytotoxic AuNCs@L-GSH can down-regulate the mRNA expression level of *TET1* and *TET*2, and also induce the aggregations of TET proteins through oxidation of their catalytic domains. These alterations can result in the decreasing of the catalytic activities of TET proteins and the subsequent global decline of 5hmC levels, which leads to gene expression changes that are involved in cell adhesion, migration, proliferation, differentiation and cell apoptosis.

Since low-cytotoxic or non-cytotoxic dosages of AuNCs have been widely used in biomedicine, our data proved that even a non-cytotoxic dose of AuNCs@L-GSH has the potential to cause epigenetic changes as analyzed by 5hmC and *TET* mRNA levels, which raise the concern about the safety associated with applications of the AuNCs in particular and nanomaterials in general. Further studies must be conducted in this field to achieve a deeper understanding of the physiological and (epi)genetic effects of AuNCs. These findings suggest that with the increasing translational applications of gold nanoparticles from the benchtop to the clinic, researchers should carefully evaluate all aspects of biosafety, especially in genetics and epigenetics.

## Methods

### Synthesis and characterization of glutathione-capped chiral AuNCs

In a typical synthesis, freshly prepared aqueous solutions of HAuCl4 (10 mM, 10 ml) and GSH (L-GSH or D-GSH; 150 mM, 2 ml) were mixed and stirred for 20 min at room temperature. The reaction mixture was then cooled down to 0 °C (ice-bath), and subsequently, tetrabutylammonium borohydride (5 equiv/mol of Au, 3 ml) was added rapidly and then stirred quickly for 4 h. A side-product of the insoluble substance was identified to be Au(I)-SG complexes^27^. The supernatant was further purified by adding three times amount of ethanol into the aqueous solution and centrifuge at 10,000 rpm for 15 min. Under such condition, the Au NCs were precipitated out of the solution while the free GSH and gold ions remained in the solution. The precipitates were then re-suspended in ultrapure water and stored at 4 °C for further experiments.

The size and morphologies of the Au NCs were characterized by High-resolution TEM (HRTEM) on JEM-2100F (JEOL, Japan) with an acceleration voltage of 200 kV. UV-vis spectra were measured with a Varian Cary 50 spectrophotometer (Varian Inc., Palo Alto, CA, USA) equipped with a 10-mm quartz cell, where the light path length was 1 cm. Photoluminescence (PL) and photoluminescence excitation (PLE) spectra of Au NCs were recorded on a Hitachi FL-4600 spectrofluorometer. Circular dichroism (CD) measurements of Au NCs were performed on a Jasco J-815 CD spectrometer (Jasco International, Tokyo, Japan) in quartz cuvettes with a path length of 10 mm at room temperature. Data were collected every 0.2 nm with a bandwidth of 1 nm, at 50 nm min^−1^ and aver- aging over three scans. Size distribution and zeta potential of Au NCs were determined by dynamic light scattering using a NICOMP 380 ZLS Zeta potential/Particle sizer (PSS Nicomp, Santa Barbara, CA, USA).

### Cell culture and cell viability assay

The human gastric cancer cell line MGC-803 cells were obtained from the Cell Resource Center, Shanghai Institute of Biochemistry and Cell Biology at the Chinese Academy of Sciences, and human embryonic kidney cell line HEK 293FT cells were purchased from Invitrogen^51^. These cells were cultured in Dulbecco’s Modified Eagle’s Medium (DMEM, Life Technologies) with 10% fetal bovine serum (Gibco), 100 U/mL penicillin and 100 μg/mL streptomycin (Life Technologies) at 37 °C in a humidified 5% CO_2_ atmosphere. Cell viability was detected by the 3-[4, 5-dimethyl-2-thiazolyl]-2, 5-diphenyl-2-H-tetrazolium bromide (MTT) assay. Briefly, cells were seeded at a density of 5 × 10^3^ cells/well in a 96-well plate and cultured overnight. The cells were incubated with various concentrations of AuNCs for 24 h. The supernatant was then removed and cells were washed once with phosphate-buffered saline (PBS) (pH 7.4). 150 μL DMEM and 15 μL MTT (0.5 mg/mL) were added to each well and incubated for 4 h at 37 °C. After removing the medium, 150 μL DMSO was added to each individual well. Following complete dissolution for 10 min at room temperature, the absorbance was measured at 570 nm using a standard micro plate reader (Scientific MultiskanMK3, Thermo Scientific, USA).

### Cell transfection, immunostaining and 5hmC staining

Cells were transfected using X-tremeGENE HP DNA transfection reagent (Roche) according to the manufacture’s protocol.

For immunostaining, HEK 293FT cells cultured on poly-L-Lysine coated glass coverslips were fixed with 4% paraformaldehyde for 15 min at room temperature and then treated with ice-cold methanol for 10 min at −20 °C. After being blocked by 15% donkey serum for 30 min, cells were incubated with primary antibody at 4 °C overnight. After incubation with the primary antibody, the cells were incubated for 1 h at room temperature with Alexa Fluor-labeled secondary antibodies (1:800; Molecular Probes, Leiden, The Netherlands). Then, the coverslips were mounted with glycerine/PBS containing 5 μg/mL DAPI for nuclei staining.

For 5hmC staining, the fixed cells were denatured in 2 M HCl at 37 °C for 20 min–30 min, then were neutralized in 100 mM Tris-HCl (pH 8.0) at 37 °C for 2 times, each time for 10 min. After neutralization, the cells were blocked in BSA with 15% donkey serum and incubated with 5-Hydroxymethylcytosine (5hmC) antibody (1:500; Active Motif) at 4 °C overnight and then stained in secondary antibodies and the coverslips were mounted as described above.

### Quantitative Real-time PCR

Total RNA was extracted using TRIZOL (Invitrogen) following the manufacturer’s protocol. The cDNAs were generated from 1 μg of total RNA using reverse transcriptase with random hexamer and oligo dT primers (Promega). Quantitative real-time PCR was performed using specific primers (List in Table S1) in a 20 μL reaction volume containing 10 μL 2 × SYBR Green Mix (GeneCopoeia) on the iQ5 (BioRad).

### Dot-blot assay

HEK 293FT cells were transfected with X-tremeGENE HP DNA transfection reagent (Roche) and pEGFP-N1 and GFP-TET1 plasmids respectively according to the manufacture’s protocol in 35 mm-diameter culture dishes. After 4 hours of transfection, different doses of AuNCs were added to the dishes at various final concentrations as follows: 0, 10, 30, 100, 200 and 500 μg/mL. Cells were harvested 48 hours after transfection. The genomic DNA was isolated using the EZNA Tissue DNA kit (OMEGA). For dot-blot assays, we followed the procedures described previously^31^. The DNA concentration was measured by NanoDrop (Thermo Scientific). Briefly, genomic DNA was spotted on nylon membrane (GE Healthcare). The membrane was baked at 80 °C and then blocked with 5% skimmed milk in TBST for 1 h, followed by the incubation with the anti-5hmC antibody (1:5,000; Active Motif) overnight at 4 °C. After washing three times with TBST, then the membranes were incubated with POD-labeled secondary antibodies (1:125,000; Roche). Detections were performed using BM Chemiluminescence Western Blotting kit (Roche). The densitometry quantification analysis of dot-blot was done by Image J software.

### ROS measurement

Cellular ROS levels were detected with dihydroethidium (DHE)^50^. Cells were incubated with 10 μM DHE at 37 °C for 30 min, and then cells were rinsed once with PBS and immediately observed under fluorescence microscope (LeicaDMI6000B, Leica Microsystems GmbH, Germany) and the photo captured were analyzed using Image J software.

### Redox-western blot

Cell lysates were prepared using high KCl lysis buffer containing 10 mMTris-HCl, pH 8.0, 140 mMNaCl, 300 mMKCl, 1 mM EDTA, 0.5% TritonX-100 and 0.5% sodium deoxycholate with complete protease inhibitor cocktail (Roche) and 20 mM N-ethylmaleimide (NEMI) to block free thiol groups. Equal amounts of proteins (30 μg) were subjected to SDS-PAGE with loading buffer with or without β-mercaptoethanol and transferred to polyvinylidene fluoride (PVDF) membranes (Roche). The membranes were treated with 1% blocking solution in TBS for 1 h, immunoblots were probed with the indicated antibodies: anti-GFP (1:5,000; AbMart, Shanghai, China), anti-Flag (1:5,000; AbMart, Shanghai, China), anti-HA (1:5,000; AbMart, Shanghai, China), anti-Prdx-SO_3_ (1:2,000; Abcam) at 4 °C overnight. Then the membranes were washed and incubated with POD-labeled secondary antibodies (1:125,000; Roche). The immunolabeled proteins were detected by BM Chemiluminescence Western Blotting kit (Roche).

### Microarray data analysis

Human transcriptome array Affymetrix Human HTA2.0 was used in this experiment. Affymetrix GeneChip Command Console (version 4.0, Affymetrix) software was used to extract raw data. Expression Console (version1.3.1, Affymetrix) software offered RMA normalization. Differentially expressed genes were screened out through the fold change. The threshold set for up- and down-regulated genes was a fold change greater than or equal to 1.5. For gene expression heat maps, the average expression across three replicates in each condition based on the probes with the maximum signal intensity per gene was calculated. Heat maps were created using Genesis 1.7.639 based on Pearson correlation coefficients of each replicate per condition. Pathway analyses were performed using Gene Set Enrichment Analysis tool v2.0.13 (GSEA2-2.0.13). The analyses were based on differentially expressed genes in each group. Differential expression was defined as multiple testing adjusted p values smaller than or equal to 0.05 and fold change greater than or equal to 1.5-fold by probes with the maximum intensity in each gene. The enrichment results with canonical pathway gene sets C2CP were reported. FDR 25% or less was used to select interesting gene sets for hypothesis generation.

### Statistical analysis

Data are expressed as mean ± SEM. Statistical comparisons between groups were conducted by unpaired Student’s t-test or one-way ANOVA followed by a Newman-Keuls comparison test. *indicates *p* < 0.05; **indicates *p* < 0.01; ***indicates *p* < 0.001. The value of *p* < 0.05 was considered to be significant.

## Additional Information

**How to cite this article**: Ma, Y. *et al*. Chiral Antioxidant-based Gold Nanoclusters Reprogram DNA Epigenetic Patterns. *Sci. Rep.* **6**, 33436; doi: 10.1038/srep33436 (2016).

## References

[b1] Dreaden E. C., Alkilany A. M., Huang X. H., Murphy C. J. & El-Sayed M. A. The golden age: gold nanoparticles for biomedicine. Chem Soc Rev 41, 2740–2779 (2012). 22109657 10.1039/c1cs15237h PMC5876014

[b2] Conde J. . Revisiting 30 years of biofunctionalization and surface chemistry of inorganic nanoparticles for nanomedicine. Front Chem. 2, 48 (2014). 25077142 10.3389/fchem.2014.00048 PMC4097105

[b3] Luo Z., Zheng K. & Xie J. Engineering ultrasmall water-soluble gold and silver nanoclusters for biomedical applications. Chem Commun (Camb) 50, 5143–5155 (2014). 24266029 10.1039/c3cc47512c

[b4] Yao Q. . Counterion-assisted shaping of nanocluster supracrystals. Angew Chem Int Ed Engl 54, 184–189 (2015). 25376748 10.1002/anie.201408675

[b5] Yao Q., Yuan X., Yu Y., Xie J. & Lee J. Y. Introducing amphiphilicity to noble metal nanoclusters via phase-transfer driven ion-pairing reaction. J Am Chem Soc 137, 2128–2136 (2015). 25584784 10.1021/jacs.5b00090

[b6] Goswami N., Zheng K. & Xie J. Bio-NCs–the marriage of ultrasmall metal nanoclusters with biomolecules. Nanoscale 6, 13328–13347 (2014). 25266043 10.1039/c4nr04561k

[b7] Zhang X. D. . Ultrasmall Au(10-12)(SG)(10-12) nanomolecules for high tumor specificity and cancer radiotherapy. Adv Mater 26, 4565–4568 (2014). 24817169 10.1002/adma.201400866

[b8] Conde J., Oliva N. & Artzi N. Implantable hydrogel embedded dark-gold nanoswitch as a theranostic probe to sense and overcome cancer multidrug resistance. Proc Natl Acad Sci USA 112, E1278–E1287 (2015). 25733851 10.1073/pnas.1421229112 PMC4371927

[b9] Conde J., de la Fuente J. M. & Baptista P. V. RNA quantification using gold nanoprobes - application to cancer diagnostics. Journal of Nanobiotechnology 8, 1–8 (2010). 20181241 10.1186/1477-3155-8-5 PMC2844353

[b10] Braz A. K. . *In situ* gold nanoparticles formation: contrast agent for dental optical coherence tomography. Journal of biomedical optics 17, 066003–066003 (2012). 22734759 10.1117/1.JBO.17.6.066003

[b11] Conde J., Bao C., Cui D., Baptista P. V. & Tian F. Antibody–drug gold nanoantennas with Raman spectroscopic fingerprints for *in vivo* tumour theranostics. Journal of Controlled Release 183, 87–93 (2014). 24704711 10.1016/j.jconrel.2014.03.045

[b12] Goswami N. . Luminescent Metal Nanoclusters with Aggregation-Induced Emission. J Phys Chem Lett 7, 962–975 (2016). 26912457 10.1021/acs.jpclett.5b02765

[b13] Pan Y. . Size-dependent cytotoxicity of gold nanoparticles. Small 3, 1941–1949 (2007). 17963284 10.1002/smll.200700378

[b14] Zhao J. Y. . Cytotoxicity of nucleus-targeting fluorescent gold nanoclusters. Nanoscale 6, 13126–13134 (2014). 25250903 10.1039/c4nr04227a

[b15] Senut M. C. . Size-Dependent Toxicity of Gold Nanoparticles on Human Embryonic Stem Cells and Their Neural Derivatives. Small 12, 631–646 (2016). 26676601 10.1002/smll.201502346 PMC5033512

[b16] Hauck T. S., Ghazani A. A. & Chan W. C. Assessing the effect of surface chemistry on gold nanorod uptake, toxicity, and gene expression in mammalian cells. Small 4, 153–159 (2008). 18081130 10.1002/smll.200700217

[b17] Russo V. E., Martienssen R. A. & Riggs A. D. Epigenetic mechanisms of gene regulation. (Cold Spring Harbor Laboratory Press, 1996).

[b18] Bird A. Perceptions of epigenetics. Nature 447, 396–398 (2007). 17522671 10.1038/nature05913

[b19] Berger S. L., Kouzarides T., Shiekhattar R. & Shilatifard A. An operational definition of epigenetics. Genes & development 23, 781–783 (2009). 19339683 10.1101/gad.1787609 PMC3959995

[b20] Robertson K. D. DNA methylation and human disease. Nat Rev Genet 6, 597–610 (2005). 16136652 10.1038/nrg1655

[b21] Szulwach K. E. . 5-hmC-mediated epigenetic dynamics during postnatal neurodevelopment and aging. Nat Neurosci 14, 1607–1616 (2011). 22037496 10.1038/nn.2959 PMC3292193

[b22] Tahiliani M. . Conversion of 5-methylcytosine to 5-hydroxymethylcytosine in mammalian DNA by MLL partner TET1. Science 324, 930–935 (2009). 19372391 10.1126/science.1170116 PMC2715015

[b23] Ito S. . Role of Tet proteins in 5mC to 5hmC conversion, ES-cell self-renewal and inner cell mass specification. Nature 466, 1129–1133 (2010). 20639862 10.1038/nature09303 PMC3491567

[b24] Lushchak V. I. Glutathione homeostasis and functions: potential targets for medical interventions. J Amino Acids 2012, 736837 (2012). 22500213 10.1155/2012/736837 PMC3303626

[b25] Singh S. & Nalwa H. S. Nanotechnology and health safety–toxicity and risk assessments of nanostructured materials on human health. J Nanosci Nanotechnol 7, 3048–3070 (2007). 18019130 10.1166/jnn.2007.922

[b26] Nel A., Xia T., Mädler L. & Li N. Toxic potential of materials at the nanolevel. Science 311, 622–627 (2006). 16456071 10.1126/science.1114397

[b27] Zhang C. . Insights into the Distinguishing Stress-induced Cytotoxicity of Chiral Gold Nanoclusters and the Relationship with GSTP1. Theranostics 5, 134 (2015). 25553104 10.7150/thno.10363 PMC4279000

[b28] Wossidlo M. . 5-Hydroxymethylcytosine in the mammalian zygote is linked with epigenetic reprogramming. Nat Commun 2, 241 (2011). 21407207 10.1038/ncomms1240

[b29] Koh K. P. . Tet1 and Tet2 regulate 5-hydroxymethylcytosine production and cell lineage specification in mouse embryonic stem cells. Cell Stem Cell 8, 200–213 (2011). 21295276 10.1016/j.stem.2011.01.008 PMC3134318

[b30] He Y. F. . Tet-mediated formation of 5-carboxylcytosine and its excision by TDG in mammalian DNA. Science 333, 1303–1307 (2011). 21817016 10.1126/science.1210944 PMC3462231

[b31] Ito S. . Tet proteins can convert 5-methylcytosine to 5-formylcytosine and 5-carboxylcytosine. Science 333, 1300–1303 (2011). 21778364 10.1126/science.1210597 PMC3495246

[b32] Iyer L. M., Tahiliani M., Rao A. & Aravind L. Prediction of novel families of enzymes involved in oxidative and other complex modifications of bases in nucleic acids. Cell Cycle 8, 1698–1710 (2009). 19411852 10.4161/cc.8.11.8580 PMC2995806

[b33] Pastor W. A., Aravind L. & Rao A. TETonic shift: biological roles of TET proteins in DNA demethylation and transcription. Nat Rev Mol Cell Bio 14, 341–356 (2013). 23698584 10.1038/nrm3589 PMC3804139

[b34] Conde J., Oliva N., Atilano M., Song H. S. & Artzi N. Self-assembled RNA-triple-helix hydrogel scaffold for microRNA modulation in the tumour microenvironment. Nat Mater 15, 353–363 (2016). 26641016 10.1038/nmat4497 PMC6594154

[b35] Hahn M. A. . Dynamics of 5-hydroxymethylcytosine and chromatin marks in Mammalian neurogenesis. Cell Rep 3, 291–300 (2013). 23403289 10.1016/j.celrep.2013.01.011 PMC3582786

[b36] Huang Y. . Distinct roles of the methylcytosine oxidases Tet1 and Tet2 in mouse embryonic stem cells. Proc Natl Acad Sci USA 111, 1361–1366 (2014). 24474761 10.1073/pnas.1322921111 PMC3910590

[b37] Tevosian S. G. . HBP1: a HMG box transcriptional repressor that is targeted by the retinoblastoma family. Genes & development 11, 383–396 (1997). 9030690 10.1101/gad.11.3.383

[b38] Audas T. E., Li Y., Liang G. & Lu R. A novel protein, Luman/CREB3 recruitment factor, inhibits Luman activation of the unfolded protein response. Mol Cell Biol 28, 3952–3966 (2008). 18391022 10.1128/MCB.01439-07 PMC2423117

[b39] Xu C., Bailly-Maitre B. & Reed J. C. Endoplasmic reticulum stress: cell life and death decisions. Journal of Clinical Investigation 115, 2656 (2005). 16200199 10.1172/JCI26373 PMC1236697

[b40] Tsai Y.-Y. . Identification of the nanogold particle-induced endoplasmic reticulum stress by omic techniques and systems biology analysis. ACS nano 5, 9354–9369 (2011). 22107733 10.1021/nn2027775

[b41] Qiu J. . Id1 induces tubulogenesis by regulating endothelial cell adhesion and cytoskeletal organization through beta1-integrin and Rho-kinase signalling. Int J Mol Med 28, 543–548 (2011). 21743954 10.3892/ijmm.2011.741

[b42] Massari M. E. & Murre C. Helix-loop-helix proteins: regulators of transcription in eucaryotic organisms. Mol Cell Biol 20, 429–440 (2000). 10611221 10.1128/mcb.20.2.429-440.2000 PMC85097

[b43] Norton J. D. ID helix-loop-helix proteins in cell growth, differentiation and tumorigenesis. J Cell Sci 113 (Pt 22), 3897–3905 (2000). 11058077 10.1242/jcs.113.22.3897

[b44] Bhattacharya R., Kowalski J., Larson A. R., Brock M. & Alani R. M. Id1 promotes tumor cell migration in nonsmall cell lung cancers. J Oncol 2010, 856105 (2010). 20414347 10.1155/2010/856105 PMC2855985

[b45] Zou J., Wang X., Zhang L. & Wang J. Iron nanoparticles significantly affect the *in vitro* and *in vivo* expression of Id genes. Chem Res Toxicol 28, 373–383 (2015). 25522732 10.1021/tx500333q

[b46] Fu H. L. . TET1 exerts its tumor suppressor function by interacting with p53-EZH2 pathway in gastric cancer. J Biomed Nanotechnol 10, 1217–1230 (2014). 24804542 10.1166/jbn.2014.1861

[b47] Zhao X. . Dynamics of ten-eleven translocation hydroxylase family proteins and 5-hydroxymethylcytosine in oligodendrocyte differentiation. Glia 62, 914–926 (2014). 24615693 10.1002/glia.22649

[b48] Thannickal V. J. & Fanburg B. L. Reactive oxygen species in cell signaling. Am J Physiol Lung Cell Mol Physiol 279, L1005–L1028 (2000). 11076791 10.1152/ajplung.2000.279.6.L1005

[b49] Zhang C. . Glutathione-capped fluorescent gold nanoclusters for dual-modal fluorescence/X-ray computed tomography imaging. Journal of Materials Chemistry B 1, 5045–5053 (2013). 10.1039/c3tb20784f 32261095

[b50] Mi Y. J. . Amino-Nogo-A antagonizes reactive oxygen species generation and protects immature primary cortical neurons from oxidative toxicity. Cell Death Differ 19, 1175–1186 (2012). 22261619 10.1038/cdd.2011.206 PMC3374081

[b51] Chen K., Mi Y. J., Ma Y., Fu H. L. & Jin W. L. The mental retardation associated protein, srGAP3 negatively regulates VPA-induced neuronal differentiation of Neuro2A cells. Cell Mol Neurobiol 31, 675–686 (2011). 21350945 10.1007/s10571-011-9664-7 PMC11498520

